# Effectiveness of a lightweight portable auto-CPAP device for the treatment of sleep apnea during high altitude stages of the Dakar Rally: a case report

**DOI:** 10.5935/1984-0063.20180023

**Published:** 2018

**Authors:** Marius Lebret, Bernard Wuyam, Dominique Bertrand, Christiane Chaudot, Jean-Louis Pépin, Jean-Christian Borel

**Affiliations:** 1 Inserm U1042, HP2 laboratory - Grenoble - Isère - França.; 2 Agir a dom., Agir a dom. - Meylan - Isère - França.; 3 Thorax and vessels division Grenoble Alpes University Hospital, Sleep Laboratory and Exercise Physiology - Grenoble - Isère - França.; 4 Pulmonary and Sleep clinic, Pulmonary and sleep clinic - St Ismier - isère - França.

**Keywords:** Altitude, Acetazolamide, Continuous Positive Airway Pressure, Sleep Apnea Syndromes

## Abstract

Sleep-related breathing disturbances are exacerbated at altitude in patients with Obstructive Sleep Apnea (OSA). The objective of this case report was to determine if a portable auto-CPAP device effectively treated sleep apnea across different altitudes. We report the severity of sleep apnea from 60 to 12,000 feet high in a man with severe OSA (Apnea Hypopnea Index at diagnosis = 60 events/hour) during the 2017 Dakar rally over the Andes mountains. The man was equipped with a lightweight portable auto-CPAP device with a narrow window [6-8 cmH_2_O]. Pressures delivered and corresponding residual events were assessed at different altitudes. The 95^th^ percentile pressure reached the maximal set pressure at the highest altitudes, and residual AHI increased from 5 events/hour to 45 events/hour at the highest altitudes. Potential mechanisms behind the development of central apnea, and optimal clinical management at altitude are discussed in the light of the findings.

## INTRODUCTION

Sleep-related breathing disturbances are exacerbated at altitude in patients with Obstructive Sleep Apnea (OSA)^1^^,^^2^. At moderate to high altitudes (~5200-8500 feet), exacerbations of obstructive apnea and hypopnea can be adequately treated with automatic continuous positive airway pressure (auto-CPAP)^3^; however central respiratory events^4^^,^^5^ cannot be controlled by auto-CPAP alone^3^.

Administration of a carbonic anhydrase inhibitor, such as Acetazolamide^6^, along with auto-CPAP is therefore recommended for altitudes above 1600m^7^. Acetazolamide stimulates ventilation by promoting renal elimination of bicarbonates, thus inducing metabolic acidosis. It attenuates post-arousal hyperventilation and loop gain following arousal, reducing central apnea^8^^,^^9^.

This paper reports sleep apnea severity in a man treated with a portable auto-CPAP device, but without acetazolamide, while he worked as a technical assistant during the 2017 Dakar Rally across the Andes Mountains. The aim was to determine if the portable auto-CPAP device (Transcend auto™ mini CPAP), designed for travel, effectively treated sleep apnea across a wide range of altitudes.

## CASE REPORT

A 57-year-old man with severe obstructive sleep apnea who had been using an auto-CPAP device (DreamStarT Auto, SEFAM, France) for 4.5 years consulted his home care provider a few weeks prior to participating in the 2017 Dakar Rally in view of obtaining a portable system. At the time of diagnosis in Grenoble, France (1000 feet) his OSA parameters were as follows: Apnea Hypopnea Index (AHI)=60 events/hour, obstructive AHI=39,3 events/hour, central AHI=4,1 events/hour, mixed AHI=13,8, mean SpO_2_=90.1% and Oxygen Desaturation Index: 61.8 events/hour; % of sleep time <90% = 35.8). At 4.5 years, the parameters were: min-max pressures=6-8 cmH_2_O, average nightly use=4.5h/night and residual AHI=7.9 events/hour).

Comorbidities included obesity (body mass index-BMI=34.9 kg/m^2^) and hypertension treated by angiotensin II antagonists, with no cardiovascular events. At Grenoble, in France (altitude <900 feet) the arterial blood gas test showed PaO_2_=86mmHg, PaCO_2_=38mmHg and pH=7.39. The exercise test showed a high ventilatory response to hypoxia (0.87 L/min/% SpO_2_/kg), i.e. above the threshold associated with an increased risk of acute mountain sickness^10^. Acetazolamide was not prescribed because potential adverse effects could not be assessed before departure.

**Figure 1:**
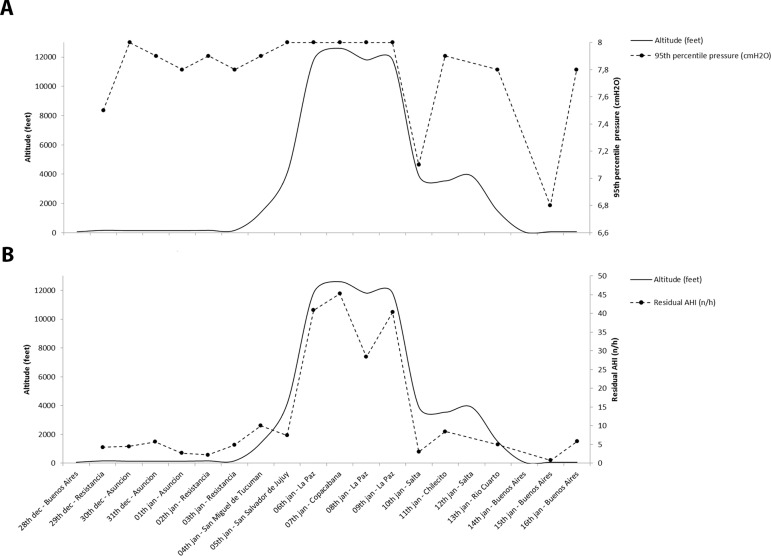
A - Altitude and 95th percentile at each stage of the trip. B - Altitude and residual AHI at each stage of the trip.

The patient was equipped with the Transcend auto™ mini CPAP device, set to his usual minimal and maximal pressures and using his usual mask (nasal); tolerance was good. He was asked to note the altitude at which he slept each night. On return to France, data from the built-in CPAP software were extracted and analyzed. Figures 1a and 1b show the variations in 95^th^ percentile pressures and the residual apnea-hypopnea index for each bivouac altitude.

The 95^th^ percentile pressure was highest at the highest altitudes (La Paz and Copacabana). The residual AHI, estimated by the built-in software, closely followed the different altitudes. Medication that could potentially impact sleep apnea severity^11^ (hypnotic, sedative drugs or opiods) were not used during the trip. Alcohol consumption was not accurately documented; the patient reported a consumption <3 units of alcohol per day.

## DISCUSSION

The latest CPAP devices are equipped with pressure compensating sensors that can compensate altitude-related drops in atmospheric pressure^12^. Manufacturers usually guarantee that devices provide adequate pressure until ~ 8200 feet. In this case report we observed that the 95^th^ percentile pressure reached the maximal set pressure at the highest altitudes (~ 13000 feet), far above the manufacturer’s guarantees.

Without the use of acetazolamide, the residual apnea-hypopnea index clearly rose as altitude increased, likely due to the development of central sleep apnea^3^. The 95^th^ percentile pressure reached the maximal pressure during the four nights at the highest altitudes compared to most other nights during which the pressure was slightly lower (Figure 1a).

Central apnea or hypopnea can occur with narrowing or closing of the upper airway^13^^,^^14^, however auto-CPAP devices may not accurately distinguish between central and obstructive events, thus resulting in inappropriate increases in pressure. Latshang et al.^3^ reported that median auto-CPAP pressure was significantly higher at 8500 feet compared to 1600 feet, however the increase in pressure due to central events did not occur when acetazolamide was used. These results suggest that when auto-CPAP is used to treat OSA at altitude, acetazolamide should be administered, or the pressure should be fixed.

Altitude conditions are also experienced during long-haul-flights. Commercial aircrafts are pressurized to cabin altitudes of up to 8000 feet. At this altitude, the partial pressure of oxygen falls to the equivalent of 15% oxygen at sea level. The guidelines for passengers with chronic respiratory diseases recommend that individuals with obstructive sleep apnea use their CPAP devices and avoid sleeping tablets, sedatives and alcohol consumption during flights^15^^,^^16^.

Use of acetazolamide during long-haul-flights may be pertinent; however this needs to be evaluated before it is recommended. For patients with severe OSA who are planning long-haul-flights, it might be useful to evaluate the combined use of CPAP and acetazolamide on a Specific Hypoxic Challenge Test (HTC) during sleep, based on a chronic intermittent hypoxia model^17^.

This case study has several limitations: first, the patient was not asked to report day to day symptoms, or physical and intellectual performance during the trip^18^. Therefore, the clinical consequences of sleep disturbances and tolerance at high altitude could not be estimated. Secondly, the patient was not asked to document his daily alcohol consumption. He reported a moderate consumption (<3 units/day) that was constant throughout the trip. Therefore, although we could not formally exclude an effect of alcohol on sleep-related respiratory events, variations of altitudes were likely the main determinant of the residual AHI. Finally, the range of set pressures on the auto-CPAP device was limited (min-max=6-8 cmH_2_O). It might have been more interesting to evaluate the response of the auto-CPAP to a broader range of pressures.
